# The pathology of *Chironex fleckeri* venom and known biological mechanisms

**DOI:** 10.1016/j.toxcx.2020.100026

**Published:** 2020-02-24

**Authors:** Melissa Piontek, Jamie E. Seymour, Yide Wong, Tyler Gilstrom, Jeremy Potriquet, Ernest Jennings, Alan Nimmo, John J. Miles

**Affiliations:** a James Cook University, Cairns, Queensland, Australia; b Australian Institute of Tropical Health and Medicine, James Cook University, Cairns, Queensland, Australia; c Centre for Molecular Development of Therapeutics, James Cook University, Cairns, Queensland, Australia; d Centre for Tropical Bioinformatics and Molecular Biology, James Cook University, Cairns, Queensland, Australia

**Keywords:** Chironex fleckeri, Antivenom, Molecular pathways, Venom processing, Venom-based omics

## Abstract

The large box jellyfish *Chironex fleckeri* is found in northern Australian waters. A sting from this cubozoan species can kill within minutes. From clinical and animal studies, symptoms comprise severe pain, welts, scarring, hypotension, vasospasms, cardiac irregularities and cardiac arrest. At present, there is no cure and opioids are used to manage pain. Antivenom is available but controversy exists over its effectiveness. Experimental and combination therapies performed *in vitro* and *in vivo* have shown varied efficacy. These inconsistent results are likely a consequence of the different methods used to extract venom. Recent omics analysis has shed light on the systems of *C. fleckeri* venom action*,* including new toxin classes that use pore formation, cell membrane collapse and ion channel modulation. This review covers what is known on *C. fleckeri* pathomechanisms and highlights current gaps in knowledge. A more complete understanding of the mechanisms of *C. fleckeri* venom-induced pathology may lead to novel treatments and possibly, the discovery of novel cell pathways, novel drug scaffolds and novel drug targets for human disease.

## Highlights

• Comprehensive review on *in vitro* and *in vivo* studies of *Chironex fleckeri* venom.
• Catalogs the usefulness of existing and experimental therapies.
• Contrasts methodologies of *Chironex fleckeri* venom processing for experimental validation.
• Details data that indicates *Chironex fleckeri* venom pathology operates through multiple mechanisms of action.
• Emphasises that venom characteristics are highly underrepresented across marine phylum.

## 1 Introduction

*Chironex fleckeri* is a large box jellyfish commonly found in Australia waters, and suspected through out the Indo Pacific region (Fenner and Williamson, 1996, Gershwin et al., 2010, Tibballs, 2006, Lippmann et al., 2011). *C. fleckeri* has one of the most lethal and rapidly active venoms known (Bailey et al., 2005, Sizemore, 1987, Tibballs, 2006; Hartwick et al., 1980). *C. fleckeri* has caused 77 known fatal stings in the last century (Currie, 1994, Keesing et al., 2016) and with the greatest risk of envenoming occurring in northern Australian waters from October through May, though fatalities have been recorded in every month of the year except July (Currie, 2003). Nearly all envenomations occurred in shallow water beaches, 92% of stings occurred between October through June (Currie, 2005).

**Fig. 1:**
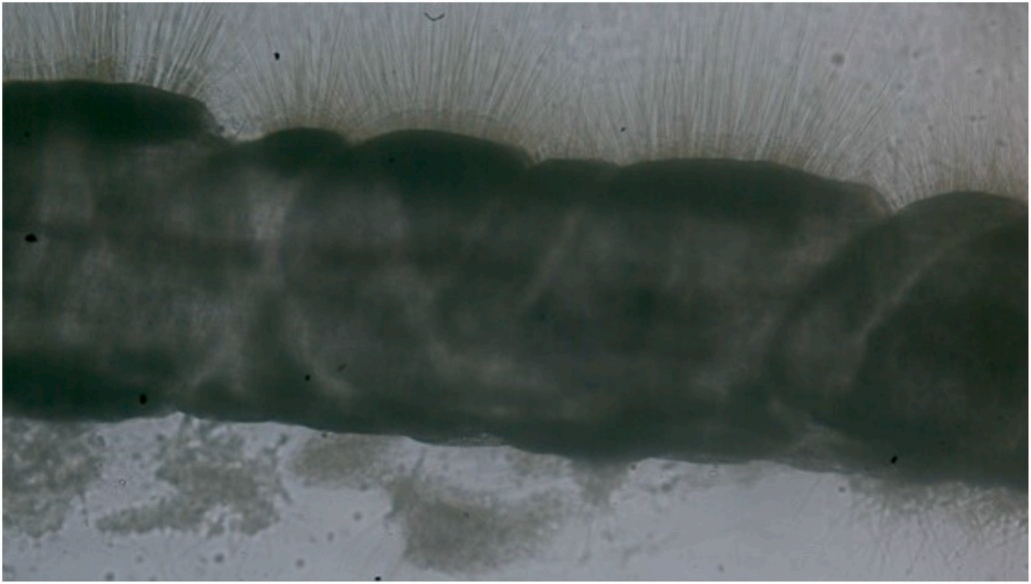
***C. fleckeri* tentacle nematocysts firing.***C. fleckeri* nematocyst spikes imaged *in vitro* using a high speed phantom VEO camera https://biopixel.tv/.

**Fig. 2:**
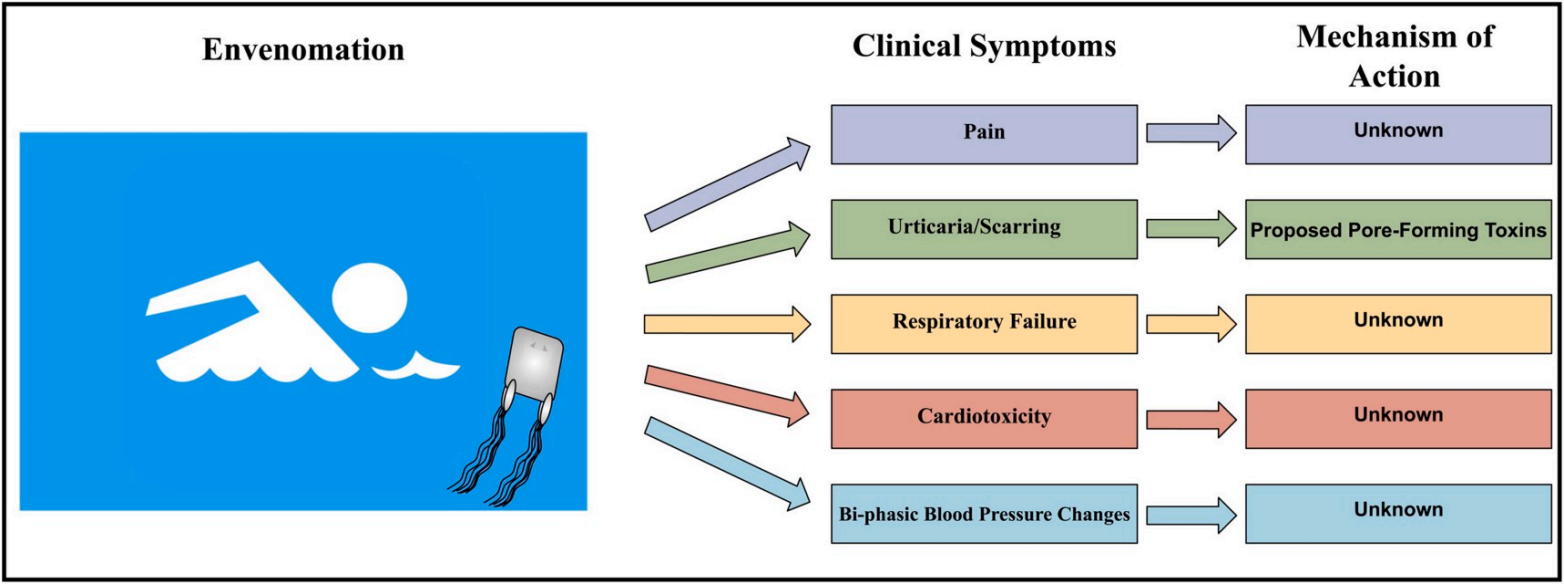
**Clinical symptoms and mechanisms underlying *C. fleckeri* envenomation.** Flow chart includes known physiological responses to *C. fleckeri* envenomation in case studies or *in vivo* and highlights unknown mechanisms of action associated with these responses.

Envenomation occurs at the epidermis, with jellyfish tentacles releasing hundreds of millions of nematocysts per sting (Fig. 1). Nematocysts are biological syringes that eject small molecules, lipids, carbohydrates, proteins and small molecules into prey with harpoon-like protrusions that may be activated upon mechanical, osmotic, chemical and neuronal reception (Kass-Simon and Scappaticci, 2002). Clinical reports of non-fatal *C. fleckeri* envenomation have contributed to the understanding of systemic venom effects including severe pain, urticaria and scarring from nematocyst contact, hypotension, vasospasms, cardiac irregularities and acute onset of cardiac arrest (Burnett et al., 1998, Cegolon et al., 2013, Nimorakiotakis and Winkel, 2003). There are no standard treatment options for *C. fleckeri* and envenomation given the pathomechanisms are unknown. A randomized controlled trial found no difference in efficacy between icepacks and hot water immersion in reducing pain (Isbister et al., 2017). Other treatments have been trialed including vinegar application, opioids and antivenom (Currie, 2005, Burnett et al., 1990) (see Fig. 2).

Given the potency and extremely rapid activity of *C. fleckeri* venom, it is interesting to note that many of these venom proteins have not been described in current gene or protein databases (Bailey et al., 2005, Brinkman et al., 2015, Brinkman et al., 2015, Burnett et al., 1996, Sizemore, 1987, Tibballs, 2006). *C. fleckeri* venom has pore-forming proteins and is considered hemolytic, it may also have ion channel modulating characteristics (Bailey et al., 2005, Burnett et al., 1996, Burnett et al., 1998, Mustafa et al., 1995). Mechanism of action studies on *C. fleckeri* venom have suggested ionophoric effects (Bailey et al., 2005, Freeman and Turner, 1969, Mustafa et al., 1995, Winter et al., 2007) and more research is needed. Given the potency and evolutionary distinctiveness of *C. fleckeri* venom, there is potential for venom-derived drug discovery and development (circulatory medicine etc.). Further study of this venom's mode of action may lead to effective standard treatments. This review summarises what is known on the mechanism of *C. fleckeri* action and highlights knowledge gaps.

## 2 Animal pathophysiology following *C. fleckeri* envenomation

Animal studies found that *C. fleckeri* venom had potent effects on the cardiovascular and pulmonary systems (Freeman and Turner, 1969, Ramasamy et al., 2005a, Ramasamy et al., 2005b, Tibballs et al., 1998). Effects included dramatic changes in arterial and venous pressure, heart rate, and abdominal respiratory physiology in anaesthetised rabbits and mice post exposure. *C. fleckeri* was extremely potent in extremely small volumes (0.1 mL of a 5,000-fold dilution of tentacle extract) and was enough to kill a mouse within 2 min (Freeman and Turner, 1969). Rabbits challenged with *C. fleckeri* venom exhibited survival times between 1 and 6 min. The physiological effects in rabbits comprised: (i) bi-phasic blood pressure changes with resting arterial pressure rising from 115 mmHg to 140 mmHg in 8 s; (ii) A plummet in arterial pressure to 225 mmHg followed a spike in 90 s; (iii) Apnea followed hypoxia; (iv) Respiratory failure and acute hypotension; (v) Bradycardia and a T-wave inversion; (vi) Arterial pressure failure and deteriorating heart rates (from 260 to 340 beats/min to 100–150 beats/min) and; (vii) Finally, terminal respiratory failure (Freeman and Turner, 1969, Freeman and Turner, 1971). These effects have been replicated in other studies (Burnett et al., 1998, Hughes et al., 2012, Tibballs et al., 1998). While the animal response to *C. fleckeri* venom has been well documented, clinical case reports are limited to describing basic symptoms such as welts on the epidermis or the cardiotoxic effects from fatalities (Currie, 2005).

## 3 The effects of antivenom on *C. fleckeri* envenomation

No one nationwide recommendation for first-aid can be made due to differences between jellyfish species. It was hoped that treatment with antivenom would prevent effects seen in severe envenomation by neutralising causative toxins in the venom. The Commonwealth Serum Laboratories (CSL) first produced *C. fleckeri* antivenom in the 1970s using hyper-immunized sheep (Baxter and Marr, 1974, Currie and Jacups, 2005) and continue to produce antivenom. However, *C. fleckeri* antivenom efficacy has been called into question through data from animals and patients (Currie, 2003, Currie and Jacups, 2005, Endean and Sizemore, 1988, Konstantakopoulos et al., 2009, Ramasamy et al., 2003, Winter et al., 2007, Winter et al., 2009).

Studies using antivenom treatment post *C. fleckeri* venom challenge have produced varying results. In one study, mice administered antivenom after *C. fleckeri* venom challenge showed 7% survival (Endean and Sizemore, 1988). However, another study showed 59% survival after *C. fleckeri* venom challenge (Burnett et al., 1990). These findings were confirmed in a related study (Winter et al., 2009). Using rat aortic smooth muscle cell line, *C. fleckeri* antivenom treatment showed no neutralisation (Konstantakopoulos et al., 2009). Interestingly, a dose study reported that the high amounts of antivenom did not improve protection in piglets (Tibballs et al., 1998) suggesting a concentration limit for efficacy. These results were confirmed in another study which showed the antivenom was effective within a limited dose range (Andreosso et al., 2014, Bloom et al., 1999).

Due to the underwhelming effects of antivenom in animal models, researchers next examined antivenom prophylactically. Prophylactic use of antivenom prolonged the survival time of mice by 5-fold (Endean and Sizemore, 1988). A separate study showed that while prophylactic administration of antivenom did not have any effect on blood pressure after envenomation, the antivenom did prevent cardiovascular collapse in 40% of test animals (Ramasamy et al., 2004). In contrast, another prophylactic study reported improved arterial pressure post low dose administration (Hughes et al., 2012, Ramasamy et al., 2004, Winter et al., 2007). In a follow up study, antivenom given 15 min before venom delivery did not prevent cardiovascular collapse and a very high dose was unable to attenuate venom effects (Winter et al., 2009). In addition, antivenom administered with magnesium sulfate could prevent cardiovascular collapse (Ramasamy et al., 2004). Collectively, prophylactic administration of antivenom shows varying effects and is impractical.

Another methodology was to combine *C. fleckeri* venom with antivenom and administer the mixture. This improved cell survival *in vitro* and improved animal survival (Andreosso et al., 2014, Konstantakopoulos et al., 2009, Tibballs et al., 1998, Winter et al., 2009). In a related study, CSL antibodies were compared with polyclonal rabbit antibodies raised against nematocyst-derived venom (Winter et al., 2009). Neither were effective therapeutically. Again, mixing *C. fleckeri* venom with antivenom is an impractical solution.

There is currently no consensus on how much antivenom is required to be effective. From animal data, effective antivenom amounts would be 3–4 fold higher than is suggested for humans (Andreosso et al., 2014, Endean and Sizemore, 1988). Two studies have reported risk of adverse reactions at low antivenom doses (Sutherland, 1977, Williamson et al., 1980) and a clinical case study in Australia reported that only 5% of patients received antivenom for *C. fleckeri* envenomation (Currie and Jacups, 2005). In addition to these data, there has been 4 fatalities even after the use of antivenom (Currie and Jacups, 2005).

## 4 Experimental and combination therapies for *C. fleckeri* envenomation

The effects of *C. fleckeri* venom on small mammalian physiology have been examined with various experimental therapeutics (Bloom et al., 1999, Burnett, 1989, Burnett et al., 1998, Freeman and Turner, 1969, Isbister et al., 2004a, Isbister et al., 2004b, Ramasamy et al., 2005a, Ramasamy et al., 2005b, Tibballs, 2006). Due to the effects on the cardiovascular and pulmonary systems, one study looked at the combined effect of antivenom with Verapamil. Verapamil is a calcium channel blocker which has been shown to relax the muscles of the heart and is used to treat high blood pressure, angina, and cardiac arrhythmias (Burnett et al., 1990, Guyton, 2011). Post injection with *C. fleckeri* venom, all untreated mice died within 7.5 min of venom administration. Here, 59% of the animals administered antivenom died within 3 min, 68% of the animals administered Verapamil died within 24 min, and 26% of mice given a mixture of antivenom and Verapamil died within 34 min (Burnett et al., 1990). Thus, Verapamil exhibited prolonged survival times. A related study attempted a rescue experiment post envenomation using a combination of antivenom and Verapamil (Bloom et al., 1999). This treatment nearly doubled the survival time.

However, addition of verapamil to a higher dose of antivenom did not increase survival time. Antivenom alone was shown ineffective. These findings were confirmed in a similar study (Winter et al., 2009).

Additional studies using Verapamil have also shown inconsistent results prophylactically and therapeutically (Burnett et al., 1990, Isbister et al., 2004a, Tibballs et al., 1998). It was additionally noted that Verapamil may cause hypotension, vasodilation and have adverse inotropic effects (Tibballs et al., 1998). However, another calcium channel blocker, Felodipine, was able to block the *C. fleckeri* venom effects *in vitro* (further detailed in section 5) (Winter et al., 2007). Collectively, the results of *C. fleckeri* venom treatment have been inconsistent both *in vitro* and *in vivo*.

## 5 Consistencies and variabilities in animal testing

Overall, the above animal studies consistently found *C. fleckeri* venom caused the following effects in mice, rats, rabbits, and piglets: (i) bi-phasic pressor responses with extreme hypertension followed by hypotension and; (ii) cardiovascular collapse and cardiotoxicity characterised by electrophysiology dysfunction with survival times as short as 1–2 min. One aspect that makes comparing results between studies difficult is varying concentrations of venom used in testing. Importantly, historical animal studies did not report venom profiles which may contribute to the variability between results. Two aspects that contribute to the venom profile are, collection site and ontogenetic variations. For instance, *C. fleckeri* venom from Weipa, Australia was found to be more lethal compared with venom taken from Mission Beach, Australia, a location nine hundred kilometres south (Brinkman et al., 2015, Winter et al., 2009). The size of the jellyfish is also relevant. Extremely potent cardiotoxic venom has been observed in large *C. fleckeri* samples (Brinkman et al., 2015, McClounan and Seymour, 2012). In future, it would be informative to examine *C. fleckeri* specimens from multiple regions across Australian waters.

The only common finding from these above experiments is the rapid action of the venom. Treatments either increased survival times (Bloom et al., 1999) or not (Burnett et al., 1990). These inconsistencies may arise from several factors that could be attributed to venom geography/profile/preparation rather than variation in methodology. The venom profile in each of these studies was not documented and likely would have varied due to different venom extraction techniques. For example, an *in vitro* and an *in vivo* study showed inconsistent results of Verapamil (Bloom et al., 1999). Another calcium channel blocker, Felodipine (T-type calcium channel blocker), significantly reduced the contractile force in isolated rat aorta (Winter et al., 2007). The most effective treatments shown thus far are all based on ionophoric mechanisms (Bailey et al., 2005, Mustafa et al., 1995, Freeman and Turner, 1969). Non-specific voltage gated calcium channels may be involved rather than L-type voltage gated calcium channels. Immunisation has been considered as a treatment for *C. fleckeri* venom (Alam and Ashraf, 2013). Whether this is practical or cost effective is unlikely.

## 6 The divergent methods of C. *fleckeri* venom extraction

Isolating pure venom from jellyfish tentacles has presented a significant challenge for researchers. The capsular organelles that deliver the venom have highly specialised anatomy and there are several classes of nematocysts (Gershwin, 2006). Researchers have attempted numerous isolation techniques. The first venom extraction method was from milked venom (Barnes, 1967). While this collection technique was aimed to imitate natural envenomation, the authors reported the resulting material was contaminated with amniotic proteins and resulted in low antivenom yields from immunized animals.

The first mechanical activation of nematocysts involved mortar and pestle grinding in ice and filtered seawater, which resulted in low venom yields (Endean et al., 1969). Subsequent mechanical disruption of nematocysts involved whole tentacle homogenisation that was centrifuged at low temperatures termed “homogenate venom extraction” (Freeman and Turner, 1969). Another technique was beachside separation of tentacle material from nematocysts. This was performed by refrigerating tentacles in sea water for 1–4 days. Nematocysts were separated from debris in solution with a fine sieve and preserved in aliquots for freezing (Bloom et al., 1998). Other mechanical extraction techniques separated the nematocysts from the tentacle and then activated the nematocysts. This technique was termed “crude venom extraction”. Yet another example was the use of sonar or high frequency sound to rupture the nematocyst capsular organelles (Burnett et al., 1998, Mustafa et al., 1995). Many early mechanical disruption techniques involved grinding nematocysts for long periods which exposed the venom to protracted raised temperatures (Endean et al., 1969). This lead to the discovery of the thermolabile nature of *C. fleckeri* venom (Bloom et al., 1998, Burnett et al., 1998, Carrette and Seymour, 2004, Endean et al., 1969).

Researchers have also explored chemical extraction. One method, soaked tentacles in ethanol for 24hr to activate nematocysts (Jouiaei et al., 2015). This extraction method allowed venom collection in one step and revealed novel proteins. However, it is likely that exposing the venom to strong alcohol would lead to protein degradation. Since heat and chemical extraction techniques rendered venom components to degradation, a cold mechanical method was developed (Carrette and Seymour, 2004). This protocol involved soaking tentacles in sea water for five days which caused nematocysts to detach from the tentacles. A strainer was then used to separate the nematocysts from the soaking solution. The strained solution was centrifuged and the nematocyst pellet was placed into a mini-glass-bead beater. The mechanical agitation of the nematocysts was repeated to allow for maximum venom yield. The process took place in a cold room to ensure nematocysts remained temperature stable throughout. The venom was then pipetted off and passed through a micro filter before experimental use. Another method used both chemical and ethanol extraction to obtain purified porin fractions of the *C. fleckeri* venom (Yanagihara and Shohet, 2012). The study used this technique *in vitro* and *in vivo* and concluded that a pore forming mechanism must be the foremost mechanism of action of *C. fleckeri* whole venom. Hydrolysis was not observed and this extraction method may not reflect the results observed in the natural setting given it alters venom compounds.

Another study aimed to investigate the mechanism responsible for the hallmark bi-phasic blood pressure response to *C. fleckeri* envenomation from two different venom samples from separate extraction techniques (Ramasamy et al., 2005b). They showed that a pure sample extracted from nematocysts versus tentacle extact had pharmacologically distinct cardiovascular effects in rodents.

These differing extraction methods have shown that *C. fleckeri* venom activity is sensitive to changes in heat, freezing, thawing and blunt-force grinding. Many methods effect venom composition and/or generate low yields (Bloom et al., 1999, Carrette and Seymour, 2004, Endean et al., 1969, Othman and Burnett, 1990, Ramasamy et al., 2005a, Ramasamy et al., 2005b, Olson et al., 1984). Thus, a significant barrier to progress is the lack of standardisation of venom extraction. A standardised method for venom extraction that has minimal-to-no impact on venom composition will allow for more robust and replicable experiments between research sites.

## 7 The ‘omics’ of *C. fleckeri* venom

The transcriptomics and proteomics of *C. fleckeri* venom is complex. Studies using SDS-page gel have identified two proteins of interest, CfTX-1 and CfTX-2 (Brinkman and Burnell, 2007). These proteins are hemolytic or pore-forming toxins that have close homology with three other box jellyfish species, *Chiropsalmus quadrigatus*, *Carybdea rastoni* and *Alatina alata* which are also known to show hemolytic properties in their venom (Brinkman and Burnell, 2007). The conserved amino acid region (TSR1) found in *C. fleckeri* and other cubozoan toxins, may be responsible for the pore forming action of these toxins (Brinkman and Burnell, 2007, Daly et al., 2014). Fractionation of *C. fleckeri* venom using size exclusion and cation exchange chromatography revealed further complexity through the identification of three additional toxins, CfTX-A, CfTX-B and CfTX-Bt. Here, CfTX-2 was referred to as Type I toxin and CfTX-1and CfTX-2 were referred to as Type II toxins (Brinkman et al., 2014). These two toxin types were determined using bioinformatics based on phylogenetic similarities. Type II toxins showed close structural homology to a 3d-Cry toxin, which are known to induce cell lysis and death through pore forming action (Brinkman et al., 2014).

Finally, a recent transcriptomic and proteomic study of C. *fleckeri* venom revealed ~20,000 predicted proteins and over 150 potential toxins by transcriptomics. Over 250 proteins were revealed by LC MS/MS with the most abundant proteins having little-to-no blast homology to known animal or plant proteins (Brinkman et al., 2015).

## 8 The varying mechanisms of action from *C. fleckeri* venom fractions

One of the first studies to provide insight into the molecular mechanism of *C. fleckeri* envenomation was a study on the effect on cardiac transmembrane potentials (Freeman, 1974). Dissections of rabbit and guinea pig hearts were perfused with two fractions of *C. fleckeri* venom obtained via size exclusion chromatography and were termed cardiotoxin and haemolytic/cardio toxin. The cardiotoxin reduced atrial action potential spike height, maximal diastolic potential and maximal rate of rise of action potential. Effects of the cardiotoxin on right atrial preparations showed sinoatrial (SA) and atrioventricular (AV) node depression, thus producing an action potential. That would undershoot normal contestations. It was suggested that these changes were the effects of increased sodium permeability in the cell. Indeed, it had been previously shown that the inhibition of depolarization of Purkinje fibers with a sodium ion channel blocker, Tetrodotoxin (TTX), would restore cardiac action potentials (Hogan and Albuquerque, 1971). TTX was trialed to attenuate the effects of *C. fleckeri* venom fractions with minor effects (Freeman, 1974). Interestingly, washing the tissue was found to reverse the cardiotoxic effects, but the cardiotoxin and haemolytic toxin fraction combination was non-reversible. Thus, it was concluded that venom fractions contained distinct proteins, and that the two fractions exerted effects by different mechanisms. A more recent study showed similar findings. *Chironex fleckeri* venom was fractionated using fast protein liquid chromatography to isolate the cardiotoxic proteins of the venom which revealed new subfractions from CfTX-1 or CfTX-2 (Chaousis et al., 2014). Three subfractions were identified comprising CTF-α, CTF-β, and CTF-γ. A cell metabolism assay was used to monitor the effects of the fractions on human skeletal muscle cell and cardiac myocyte metabolism. This study found that CTF-α and, CTF-β were cytotoxic to cardiac myocytes and not skeletal muscle cells. CTF-α and CTF-β were observed to have a synergistic effect causing faster and permanent cell detachment and death than either toxin fraction alone. The third fraction, CTF-γ, was toxic to muscle cells but not cardiomyocytes. Since the study observed cellular recovery after 3 h, they concluded their findings did not align completely with a pore forming mechanism of action (Chaousis et al., 2014).

## 9 Ionophoric action as a potential mechanism of action for *C. fleckeri* venom activity

Interest in the cardiotoxic molecular mechanism of *C. fleckeri* venom led to an *in vitro* study on the contractile force of isolated cardiac cells and membrane potentials (Mustafa et al., 1995). The administration of *C. fleckeri* whole venom led to large increased intracellular calcium influx which caused spontaneous contractions, a decrease in developed force and an increase in resting force. Since calcium is vital for atrial contraction, calcium ion channels were thought to be responsible for this intense cardiac response. The response of papillary muscle contractility was not attenuated with the L-type calcium channel blockers Nifedipine, TTX or the sarcoplasmic reticulum inhibitor (Ryanodine). The isolated myocytes increased intracellular sodium concentrations preceding a large calcium influx. The study then preincubated papillary muscles with Ni^2+^ to block *C. fleckeri* venom effects. Ni^2+^ significantly attenuated the effect of *C. fleckeri* venom. This result led to the hypothesis that *C. fleckeri* venom could be acting through the activation of an Na^+^/Ca^2+^ exchanger. The study concluded that it was unlikely that the increase in Ca^2+^ was due to nonspecific membrane damage given the effects appeared so quickly after venom exposure.

An early study of *C. fleckeri* venom used anaesthetised Wistar rats (*Rattus norvegicus*) to monitor arterial and venous pressure, heart rate and abdominal respiratory movements (Freeman and Turner, 1969). The cause of death was from cardiotoxicity which was supported by interference with repolarization and conduction of atrial action potentials. It was reported that the toxin was haemolytic. Elevated plasma potassium was not consistently high enough to be related to the cardiac irregularities (Southcott and Kingston, 1959). The study concluded that the venom likely alters membrane permeability (Freeman and Turner, 1969). A more recent study used isolated rat aorta to measure the vascular effects of *C. fleckeri* venom (Winter et al., 2007). A venom titration produced a concentration independent sustained contraction of the endothelium-denuded aortic tissue. This study attempted to block the effects of the venom with Felodipine, which significantly reduced coronary flow velocity. Due to the positive effects of Felodipine treatment *in vitro*, the study speculated an ionophoric mechanism of action may be responsible for the effects of *C. fleckeri* venom.

Further studies on the molecular mechanism of *C. fleckeri* venom sought to confirm the increase of cytosolic calcium in myocytes as a cause for cardiotoxicity (Bailey et al., 2005). Here, cardiac ventricular myocytes were isolated and treated with a Indo-1 Ca^2+^ indicator dye before the addition of *C. fleckeri* venom. Cells pre-incubated with either lanthanum chloride or Verapamil prior were then exposed to *C. fleckeri* venom (Bailey et al., 2005). *Chironex fleckeri* venom caused an increase in free cytosolic Ca^2+^. *C. fleckeri* venom applied to myocytes preincubated with Verapamil resulted in an increase in free cytosolic Ca^2+^. *C. fleckeri* venom applied to preincubated myocytes with lanthanum caused an increase in free cytosolic Ca^2+^. Thus, the study showed that *C. fleckeri* venom did cause an increase in cytosolic calcium levels. To further investigate the source responsible for increased cytosolic calcium ions (i.e. intracellular or extracellular stores of calcium), manganese (Mn^2+^) Fura-2 quench reactions were performed. Mn^2+^ passes through the cell membrane using the same ion channels as Ca^2+^. The Mn^2+^ experiment determined that there was a notable permeability of Ca^2+^ into the myocytes under resting conditions. Venom dramatically increased the rate of extracellular Mn^2+^ influx into the cell. Thus, the increase in cytosolic Ca^2+^ is likely originating from the extracellular fluid via ion channels (Bailey et al., 2005). This was an important finding as it was previously thought the sarcoplasmic reticulum was the source of excess cytosolic calcium (Mustafa et al., 1995). Overall, several studies have proposed that different fractions of *C. fleckeri* venom have different mechanisms of action (Freeman, 1974; Chaousis et al., 2014; Ramasamy et al., 2005b). While the established mechanism for *C. fleckeri* venom pathology is proposed to be ionophoric-based (Bailey et al., 2005, Freeman and Turner, 1969, Mustafa et al., 1995, Winter et al., 2007), the exact voltage gated ion channels involved are unknown. Further research is required to confirm that calcium enters from the cell versus the extra cellular space (Bailey et al., 2005).

Three studies have tested three compounds (Nifedepine, Verapamil and Felodipine) all which were voltage gated calcium channel blockers. Only one compound (Nifedepine) was unable to attenuate *C. fleckeri* venom response (Mustafa et al., 1995). Nifedepine inhibits L-type channels but does block other members of the calcium ion channels families. Of these calcium channel blockers, only Verapamil has been tested in human envenomation (Currie, 2005). Felodipine has been tested *in vitro* and was shown to have some efficacy (Winter et al., 2007).

Finally, a very recent study also suggests that *C. fleckeri* venom likely has several more mechanisms of action (Lau et al., 2019). Using a genome-scale CRISPR knockout (GeCKO) screen, plasma membrane lipids were identified as key components in the cytotoxicity mechanism. Through the use of methyl-β-cyclodextrin or 2-hydroxypropyl-β-cyclodextrins, which deplete cholesterol from the cell membrane, researchers were able to attenuate the venom response in human cell lines. Treatment with cyclodextrins attenuated both necrosis and pain when administered after venom in animal models.

## 10 Future directions and conclusion

Animal studies have laid the ground work for understanding the mechanism of action of *C. fleckeri* venom. Across the literature, treatment and survival are highly variable in animal experiments. Improvements could be made in the future by adopting a standardisation of venom preparation as previous described (Carrette et al., 2002). This would be key in facilitating more robust conclusions across different sites. Future studies should also report the venom profile by SDS, HPLC, FPLC or LC/MS/MS for quality control. Additionally, since cardiotoxicity is well characterised by ion channel disruption, monitoring the effects of the venom on the ion channels of primary afferent (pain sensing) neurons may be a logical place for future studies to begin characterizing the pain aspect of envenomation. Another consideration for future studies would be to use *C. fleckeri* venom to map pain pathways, as severe pain is a hallmark clinical symptom.

As described above, it has been reported that *C. fleckeri* antivenom is ineffective *in vivo*. Additionally, it remains unclear if *C. fleckeri* antivenom can mitigate clinical symptoms. A number of studies have found that the antivenom may opsonize the venom if premixed. However, these approaches are not practical. The venom extraction studies agreed that *C. fleckeri* venom was susceptible to heat and (Carrette and Seymour, 2004, Olson et al., 1984). The varying conditions in venom extraction can cause active proteins to denature and/or aggregate dramatically altering the venoms’ potency (Endean et al., 1969, Othman and Burnett, 1990). Future studies should use the most non-invasive extraction technique that minimizes venom exposure to temperature changes and severe mechanical disruption. The cold mechanical extraction technique meets these requirements.

Additional omics work is needed. A full genome *C. fleckeri* sequence analysis has yet to be performed, nor detailed analysis of PTMs, small molecules, lipids or polysaccharides. Finally, marine phyla are highly underrepresented in current protein and nucleotide databases. More information is needed given the species diversity in Earth's oceans.

To better the understand mechanism of action of *C. fleckeri* venom, further ion channel physiology experiments are required. Future research should utilize new high-throughput techniques (Inserra et al., 2017, Vetter et al., 2012). *In vitro* treatments that attenuate *C. fleckeri* venom activity have been based on the assumption of ionophoric activity. Since calcium ion conductance from the extracellular fluid has been confirmed to occur upon *C. fleckeri* venom exposure, future research should investigate the involvement of the other voltage gated calcium ion channels (Freeman, 1974).

In conclusion, the studies have contributed invaluable information on the mechanism of action of *C. fleckeri* venom and has revealed areas where future work is needed. Animal experiments have been crucial in understanding the mammalian physiological response. Antivenom studies have given key insights to the rapid kinetics of *C. fleckeri* venom and have highlighted that more work is needed to improve efficacy. Venom extraction studies have helped emphasise data inconsistencies across sites and have highlighted the need for a standardised protocol for venom extraction and profiling. Omics and CRISPR studies are beginning to allow a very comprehensive look on mechanism of action. Collectively, these studies will assist in the development of novel treatments for *C. fleckeri* envenomation and potentially, the discovery of novel cell pathways and scaffolds for next generation pharmaceuticals (Jimenez et al., 2018).

## Funding

This work was supported by the 10.13039/501100000925NHMRC Project APP1108064. JJM is supported by a 10.13039/501100000925NHMRC Career Fellowship Level 2 APP1131732. This work was also supported by the Lions Foundation.

## Author contributions

MP, YW, TG, JP, JES, EJ, AN and JJM wrote and revised the review.

## Declaration of competing interest

The authors declare no competing financial or personal interests.
